# Efficient reduction of synthetic mRNA induced immune activation by simultaneous delivery of B18R encoding mRNA

**DOI:** 10.1186/s13036-019-0172-5

**Published:** 2019-05-09

**Authors:** Tatjana Michel, Sonia Golombek, Heidrun Steinle, Ludmilla Hann, Ana Velic, Boris Macek, Stefanie Krajewski, Christian Schlensak, Hans Peter Wendel, Meltem Avci-Adali

**Affiliations:** 1 Department of Thoracic and Cardiovascular Surgery, University Hospital Tübingen, Tübingen, Germany; 2 Proteome Center Tübingen, Interfaculty Institute for Cell Biology, University of Tübingen, Tübingen, Germany

**Keywords:** Immune response, B18R, Modified mRNA, Type I IFN

## Abstract

The application of synthetic modified messenger RNA (mRNA) is a promising approach for the treatment of a variety of diseases and vaccination. In the past few years, different modifications of synthetic mRNA were applied to render the mRNA more stable and less immunogenic. However, the repeated application of synthetic mRNA still requires the suppression of immune activation to avoid cell death and to allow a sufficient production of exogenous proteins. Thus, the addition of type I interferon (IFN) inhibiting recombinant protein B18R is often required to avoid IFN response. In this study, the ability of B18R encoding mRNA to prevent the immune response of cells to the delivered synthetic mRNA was analyzed. The co-transfection of enhanced green fluorescent protein (eGFP) mRNA transfected fibroblasts with B18R encoding mRNA over 7-days resulted in comparable cell viability and eGFP protein expression as in the cells transfected with eGFP mRNA and incubated with B18R protein. Using qRT-PCR, significantly reduced expression of interferon-stimulated gene Mx1 was detected in the cells transfected with B18R mRNA and stimulated with IFNβ compared to the cells without B18R mRNA transfection. Thereby, it was demonstrated that the co-transfection of synthetic mRNA transfected cells with B18R encoding mRNA can reduce the IFN response-related cell death and thus, improve the protein expression.

## Introduction

During the last few years, synthetic messenger RNA (mRNA) has gained great interest as a therapeutic agent. The synthetic mRNA-based therapies promise new opportunities for the treatment of different diseases by the induction of functional protein expression in desired cells [1–4]. Synthetic mRNA-based therapy has major advantages compared to retroviral gene therapy: (i) the mRNA does not need to enter the nucleus for translation [4–6], (ii) the translation of the mRNA takes place under physiological conditions in the cytosol, (iii) the desired proteins can be produced without integration into the genome [7, 8], and (iv) the expression of proteins by the exogenously delivered synthetic mRNAs is transient [9].

To increase the translation and stability of synthetic mRNAs, different types of modifications can be introduced during the in vitro transcription (IVT) [10, 11]. A poly-(A)-tail is attached to the 3′-end to enhance the stability and translation of synthetic mRNA [12]. In addition, a synthetic cap analog, such as the anti-reverse cap analog (ARCA, 3′-O-Me-m7G(5′)ppp(5′)G RNA cap structure analog), can be used to further increase the mRNA stability and translation efficiency. In ARCA, the 3′-OH of the m^7^G moiety is substituted by a 3′-O-methyl group, which enables the incorporation of the cap analog in the correct orientation at the 5′-end during the IVT [13]. Thereby, the mRNA degradation is prevented, the translation efficiency is improved, and the immune activation is reduced [14, 15]. Furthermore, the incorporation of modified nucleosides like 5-methylcytidine (m5C) and pseudouridine (Ψ) during the IVT into the synthesized mRNA enhances on the one hand the expressed protein level [16–18] and biological stability [16] and on the other hand suppresses the activation of the immune system [16, 19, 20].

However, in spite of these modifications, the exogenously delivered synthetic mRNA has still the potential to induce an immune activation in the cells. Pattern recognition receptors (PRRs), such as the Toll-like receptors (TLRs) 3, 7, 8 [21, 22] or the retinoic acid inducible gene I (RIG-I) [23], are able to recognize foreign RNAs inside the cells, which subsequently lead to an immune response. Thus, the recognition of exogenously delivered synthetic mRNA can lead to the activation of nuclear factor κB (NF-κB) in the cells and result in expression of type I interferons (IFNs) and proinflammatory cytokines [21, 22, 24–27]. Interferon-α (IFNα) and interferon-β (IFNβ) are the effector molecules that together form type I IFNs and mediate immune responses in cells. Thereby, defense mechanisms are activated, which lead to the depletion of the foreign RNA and inhibit the translation of mRNAs [28, 29].

Type I IFNs bind to the transmembrane interferon receptors on the cell surface and induce an antiviral state in the cells, which naturally inhibits the virus replication and reduces viral spread. The binding to the interferon receptor leads to the activation of cytoplasmic signal transductors and activators of transcription (STATs). Subsequently, the transcription of IFN-stimulated genes (ISGs) is induced in the nucleus. The products of these ISGs have numerous antiviral effector functions [29, 30]. One of the antiviral proteins encoded by ISGs is the interferon-induced GTP-binding Mx1 protein. The Mx1 protein is a GTPase, which is responsible for a specific antiviral response. Thereby, viral infections are inhibited by blocking viral transcription and replication [31, 32]. However, viruses also developed various strategies to escape this antiviral response. For example, the vaccinia virus encoded B18R protein functions as a soluble receptor for IFNα and IFNβ. This protein can exist as a soluble extracellular as well as a cell surface bound protein [33] and has a high affinity for type I IFNs. Thus, the binding of the B18R protein can block the autocrine and paracrine function of type I IFNs. Furthermore, it can also bind to the cell surface of uninfected and infected cells [34], and thereby reduce the inflammatory signal.

The repeated transfection of cells with synthetic mRNA and the following induction of IFNs result in a rapid decrease of cell viability [35, 36]. Therefore, if the delivery of synthetic mRNA is required over an extended period, such as for the reprogramming of cells into induced pluripotent stem cells (iPSCs), recombinant B18R protein can be applied to avoid the immune activation of cells and to block the activity of type I IFNs. Thus, in previous studies, the addition of recombinant B18R protein during the long-term cell reprogramming experiments with synthetic mRNAs led to an increased cell viability and a successful reprogramming of cells into iPSCs [37–39].

**Fig. 1:**
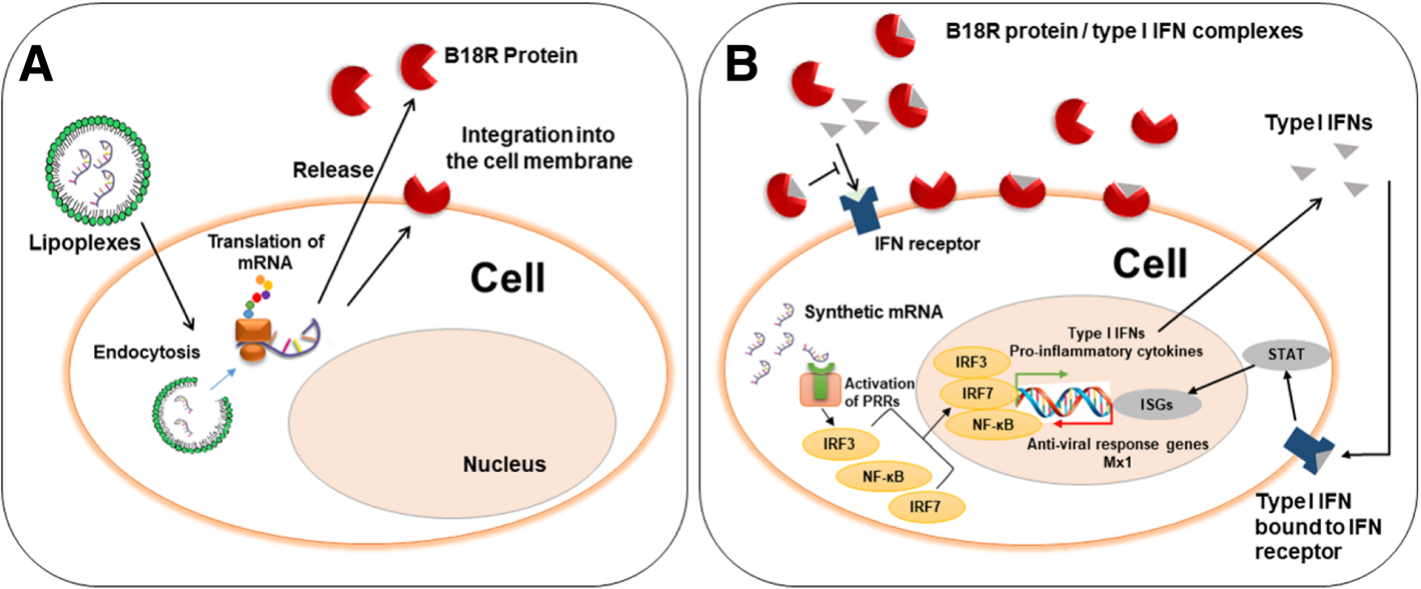
Schematic representation of the synthetic B18R mRNA transfection and translation process and the inhibition of type I interferon (IFN) immune response. (**a**) Lipoplexes are generated by complexing the B18R encoding mRNA with Lipofectamine^®^ 2000. After the uptake of lipoplexes into the cells by endocytosis and the subsequent endosomal escape, the synthetic B18R mRNA is translated by the ribosomes into functional B18R protein. Then, B18R is released into the supernatant or integrated into the cell membrane. (**b**) The recognition of synthetic mRNA by pattern recognition receptors (PRRs) in the cells leads to the activation of NF-κB, IRF3, and IRF7, which results in the expression of pro-inflammatory cytokines, e.g. type I IFNs. The produced type I IFNs bind to the IFN receptors and induce the expression of interferon-stimulated genes (ISGs), such as Mx1. The synthesized B18R proteins can capture type I IFNs produced by mRNA transfected cells, and thereby, reduce the inflammatory reaction

In this study, the effectivity of synthetic B18R mRNA co-delivery into cells along with the exogenously delivered mRNA encoding the desired protein was analyzed in order to simultaneously suppress synthetic mRNA induced immune activation in cells. The strategy of synthetic B18R mRNA delivery-based reduction of type I IFN response is presented in Fig. 1.

## Materials and methods

### Synthesis of modified mRNA

#### Amplification of plasmid inserts and adding of poly-T-tail by polymerase chain reaction (PCR)

The pcDNA 3.3 vector containing the coding sequence (CDS) of B18R (Aldevron, Fargo, North Dakota, US) or enhanced green fluorescent protein (eGFP) (Addgene, cat. no. 26822) [37] was used as template. To amplify the CDS of B18R and eGFP, the Hotstar HiFidelity Polymerase Kit (Qiagen, Hilden, Germany) was used in accordance with the manufacturer’s instructions. For the PCR, 100 ng plasmid DNA, 0.7 μM of the forward primer, 5′-TTGGACCCTCGTACAGAAGCTAATACG-3′ and 0.7 μM of the reverse primer, T_120_-CTTCCTACTCAGGCTTTATTCAAAGACCA-3′ (Ella Biotech, Martinsried, Germany), were used. PCR was performed using the following cycling protocol: initial activation step at 95 °C for 5 min, followed by 25 cycles of denaturation at 95 °C for 45 s, annealing at 58 °C for 1 min, extension at 72 °C for 1 min, and final extension at 72 °C for 5 min. After the DNA amplification, PCR products were purified using QIAquick PCR purification kit (Qiagen) and eluted in 2 × 20 μl nuclease-free water (Qiagen). The quality and purity of the DNA were assessed by 1% agarose gel electrophoresis.

#### IVT

The IVT of the DNA into mRNA was performed using MEGAscript^®^ T7 Kit (Life Technologies, Darmstadt, Germany) according to the manufacturer’s instructions. Therefore, 40 μl IVT reaction mixture containing 7.5 mM ATP, 1.875 mM GTP (both from MEGAscript^®^ T7 Kit), 7.5 mM m5C (TriLink BioTechnologies, San Diego, USA), 7.5 mM Ψ (TriLink BioTechnologies), 2.5 mM ARCA (New England Biolabs, Frankfurt am Main, Germany), 40 U RiboLock RNase inhibitor (Thermo Fisher Scientific, Waltham, USA), 1.5 μg PCR product, 1x reaction buffer and 1x T7 RNA polymerase enzyme mix was prepared. The mixture was incubated for 4 h at 37 °C, then 1 μl TURBO DNase (from MEGAscript^®^ T7 Kit) was added to the IVT reaction mixture and incubated for 15 min at 37 °C to remove the template DNA. After the incubation, mRNA was purified using RNeasy Mini Kit (Qiagen) according to the manufacturer’s instructions and eluted in 2 × 20 μl nuclease-free water. Subsequently, dephosphorylation was performed with 10 U Antarctic phosphatase (New England Biolabs) at 37 °C for 30 min. The mRNA was purified and eluted in 50 μl nuclease-free water using RNeasy Mini Kit. The concentration was measured using ScanDrop spectrophotometer (Analytic Jena, Jena, Germany) and adjusted to 100 ng/μl by adding nuclease-free water. The quality and purity of the synthesized and modified mRNA were confirmed in a 1% agarose gel. The modified mRNA was stored at − 80 °C and used for transfections.

### Cultivation of cells

BJ human foreskin fibroblasts (Stemgent, Cambridge, USA) were cultivated in DMEM with high glucose containing 10% fetal calf serum (FCS), 2 mM L-glutamine, 1% penicillin/streptomycin, and 30 mM HEPES. Cell culture medium and supplements were obtained from Thermo Fisher Scientific (Waltham, USA). Cells were kept at 37 °C with 5% CO_2_ and medium was changed every 3 days. Cells were passaged using trypsin/EDTA (0.04%/0.03%, PromoCell, Heidelberg, Germany).

### Transfection of fibroblasts with synthetic modified mRNA

To perform transfection of cells, 1 × 10^5^ fibroblasts were seeded per well of a 6-well plate and cultivated overnight at 37 °C and 5% CO_2_. Next day, the mRNA transfection of cells was performed. For the transfection, 1 ml Opti-MEM I reduced serum medium (Life Technologies, Darmstadt, Germany), 4 μl Lipofectamine^®^ 2000 (Thermo Fisher Scientific) and 1.5 μg eGFP mRNA or 1.5 μg eGFP mRNA and 0.2 to 1.5 μg B18R mRNA were mixed and incubated for 20 min at room temperature (RT) to form lipoplexes. The cells were washed with DPBS w/o Ca^2+^/Mg^2+^ (Thermo Fisher Scientific) and incubated with the transfection complexes for 4 h at 37 °C and 5% CO_2_. After the incubation, the transfection mixture was replaced by cell culture medium.

### Detection of B18R protein in B18R mRNA transfected fibroblasts using mass spectrometry (MS)

Since no commercially available antibody against B18R is available, proteome analysis was performed using MS to detect the successful production of B18R in the cells after the transfection with B18R mRNA.

#### Isolation of proteins

Fibroblasts were transfected with 1.5 μg B18R mRNA and after 24 h, cells were rinsed with cold DPBS and lysed on ice using 200 μl of Pierce™ RIPA buffer (Thermo Fisher Scientific) containing 1x Halt™ Protease Inhibitor Cocktail (Thermo Fisher Scientific) for 5 min. After the sonification for 1 min at RT in an ultrasonic bath, cell lysates were centrifuged at 14.000 g for 25 min at 4 °C. Supernatants were collected and stored at − 80 °C. Protein concentrations were determined using Pierce™ BCA Protein Assay kit (Thermo Fisher Scientific) and the microplate reader EON Synergy 2 BioTek instruments (Bad Friedrichshall, Germany).

#### SDS-PAGE and Coomassie blue staining

From the obtained protein sample, 20 μg of protein was mixed with 1x Laemmli sample buffer (Bio-Rad, Munich, Germany), denatured for 5 min at 95 °C, and separated by using 10% SDS-PAGE at 200 V for 25 min. The gel was incubated in a fixation solution composed of 50% ethanol and 10% acetic acid in ddH_2_O for 30 min at RT. Then, the gel was stained with Coomassie Brilliant Blue R-250 staining solution (Bio-Rad) for 10 min at RT, destained in 7.5% acetic acid and 25% methanol in ddH_2_O for 1 h at RT and stored in ddH_2_O overnight. Afterwards, a single band at around 42 kDa and additionally all bands from around 25 to 100 kDa were cut out and used for MS analysis.

#### Proteome analysis

Tryptic digestion of proteins: For proteome analysis, gel pieces were digested as described previously [40].

Liquid Chromatography (LC)-MS/MS: LC-MS/MS analyses were performed on an EasyLC nano-HPLC (Proxeon Biosystems) coupled to an LTQ Orbitrap Elite (Thermo). Separations of the peptide mixtures were done on a 15 cm fused silica emitter of 75 μm inner diameter (Proxeon Biosystems), in-house packed with reversed-phase ReproSil-Pur C18-AQ 3 μm resin (Dr. Maisch GmbH). Peptides were injected with solvent A (0.5% acetic acid) at a flow rate of 500 nl/min and separated at 200 nl/min. The separation was performed using a linear 75 min gradient of 5–33% solvent B (80% ACN in 0.5% acetic acid). LTQ Orbitrap Elite was operated in the positive ion mode. Precursor ions were acquired in the mass range from m/z 300 to 2000 in the Orbitrap mass analyzer at a resolution of 120,000 followed by MS/MS spectra acquisition. The 15 most intense precursor ions from the full scan were sequentially fragmented.

MS Data Processing and Analysis: Acquired MS spectra were processed with MaxQuant software package version 1.5.2.8 [41, 42], integrated with Andromeda search engine. Database search was performed against a human database obtained from Uniprot taxonomy ID 9606, containing 93,827 protein entries and 247 commonly occurring laboratory contaminants. Additionally, we used also the viral database for vaccinia virus Uniprot taxonomy 10,245, containing 5023 protein entries. Trypsin was fixed as the protease with a maximum missed cleavage of two. Oxidation of methionines and N-terminal acetylation were specified as variable modifications. Initial maximum allowed mass tolerance was set to six ppm. Carbamidomethylation on cysteines was defined as a fixed modification. Re-quantify was enabled and a false discovery rate of 1% was applied at the peptide and protein level.

### Quantitative real-time polymerase chain reaction (qRT-PCR) analyses of Mx1 expression to evaluate the type I IFN reaction inhibiting effect of B18R mRNA delivery

The interferon-induced GTP-binding protein Mx1 is expressed in the cells after the binding of IFNs to the IFN receptors on the cell surface [30]. To measure the type I IFN reaction inhibiting effect of B18R mRNA by production of B18R protein, the Mx1 mRNA expression level in the B18R mRNA transfected cells was determined. Therefore, 1 × 10^5^ fibroblasts were seeded per well of a 6-well plate. Next day, cells were transfected with 1.5 μg B18R mRNA and incubated for 4 h at 37 °C and 5% CO_2_. Afterwards, the transfection mixture was replaced by cell culture medium and the cells were incubated for 24 h at 37 °C. Subsequently, the cells were incubated for 3 h at 37 °C and 5% CO_2_ with 5 ng/ml recombinant human IFNβ (PeproTech, Hamburg, Germany).

#### Isolation of RNA and cDNA synthesis

After stimulation with 5 ng/ml recombinant human IFNβ, fibroblasts were rinsed twice with DPBS. Total RNA was extracted using the Aurum Total RNA Mini Kit (Bio-Rad) according to the manufacturer’s instructions. Then, cDNA was generated from 200 ng total RNA using iScript™ cDNA Synthesis Kit (Bio-Rad) with following conditions: 5 min at 25 °C, 30 min at 42 °C, and 5 min at 85 °C. The synthesized cDNA was diluted 1:10 for qRT-PCR.

#### qRT-PCR

The qRT-PCR was performed using iQ SYBR Green Supermix (Bio-Rad) according to the supplier’s recommendations. The reactions were run in triplicates in an iCycler iQ Real-Time PCR detection system (Bio-Rad). The expression of the constitutively expressed gene glyceraldehyde 3-phosphate dehydrogenase (GAPDH) served as an internal control for the amount of RNA input. Initial cDNA denaturation was performed at 95 °C for 3 min, followed by 40 cycles of denaturation at 95 °C for 15 s, annealing at 63 °C for 30 s, extension at 72 °C for 10 s. Levels of mRNA were normalized to GAPDH and the results are shown relative to control mRNA levels in samples treated with medium containing Lipofectamine^®^ 2000. The primers used for the specific amplification of transcripts were ordered from Ella Biotech (Martinsried, Germany). For the detection of Mx1 transcripts, the forward primer 5′-AGACAGGACCATCGGAATCT-3′ and the reverse primer 5′-ACGTCCACAACCTTGTCTTC-3′ were used, and GAPDH transcripts were detected using the forward primer 5′-TGAACCACCAACTGCTTAGC-3′ and the reverse primer 5′-GGCATGGACTGTGGTCATGAG-3′.

### Co-transfection of cells simultaneously with synthetic eGFP and B18R mRNA

#### Analysis of cell viability

To investigate the impact of synthetic B18R mRNA co-transfection on the cell viability, 1 × 10^5^ fibroblasts were seeded per well of 6-well plate. After 24 h, daily transfection of fibroblasts with 1.5 μg eGFP mRNA with or without 1.5 μg B18R mRNA was performed. Furthermore, cells transfected with 1.5 μg eGFP mRNA were cultivated without or with 200 ng/ml B18R protein (Stemgent, Lexington, USA). After each transfection, cells were incubated 24 h with the cell culture medium with or without B18R protein. Subsequently, cells were washed three times with DPBS and 300 μl RPMI without phenol red (Life Technologies) containing 0.5 μg/ml MTT (3-(4,5-dimethylthiazol-2-yl)-2,5-diphenyltetrazoliumbromide, AppliChem, Darmstadt, Germany) was added to the cells. After 4 h of incubation at 37 °C and 5% CO_2_, the medium was removed and the formazan products were solubilized by adding 300 μl of dimethyl sulphoxide (DMSO, SERVA Electrophoresis, Heidelberg, Germany) to each well of the 6-well plate for 10 min at 37 °C. The absorbance was measured at 540 nm using a microplate reader (Mithras, Bad Wildbach, Germany).

#### Analysis of eGFP expression

To analyze the eGFP expression in cells after the co-transfection with eGFP and B18R encoding mRNA, fibroblasts were seeded with the density of 1 × 10^5^ cells per well of the 6-well plate. The transfection complexes were formed by mixing 1.5 μg of eGFP mRNA and 0.2, 0.5, 1, or 1.5 μg B18R mRNA with 4 μl Lipofectamine^®^ 2000 in 1 ml Opti-MEM. After the incubation for 20 min at RT, transfection complexes were added to the cells and incubated for 4 h at 37 °C and 5% CO_2_. Afterwards, the transfection mixture was replaced by cell culture medium. The expression of eGFP was analyzed 24 h after the first transfection and 24 h after the second transfection using flow cytometry and fluorescence microscopy. For the flow cytometry, cells were detached, washed with DPBS and fixed in 1x CellFIX (BD Bioscience, Heidelberg, Germany). The flow cytometry measurements were performed using FACScan (BD Bioscience). The fluorescence microscopy was performed using Zeiss Axio Microscope (Zeiss, Oberkochen, Germany).

### Statistical analysis

Data are shown as means ± standard error of the mean (SEM). Kolmogorov-Smirnov test was used for the normality test. Normally distributed data were analyzed using one-way or two-way analysis of variance (ANOVA). Samples with non-normal distribution were analyzed using the Kruskal-Wallis test. For all statistical analysis, the software GraphPad Prism (version 6, GraphPad Software, La Jolla, USA) was used. Statistical significance was defined as *p* < 0.05.

## Results

### Production of synthetic B18R mRNA

**Fig. 2:**
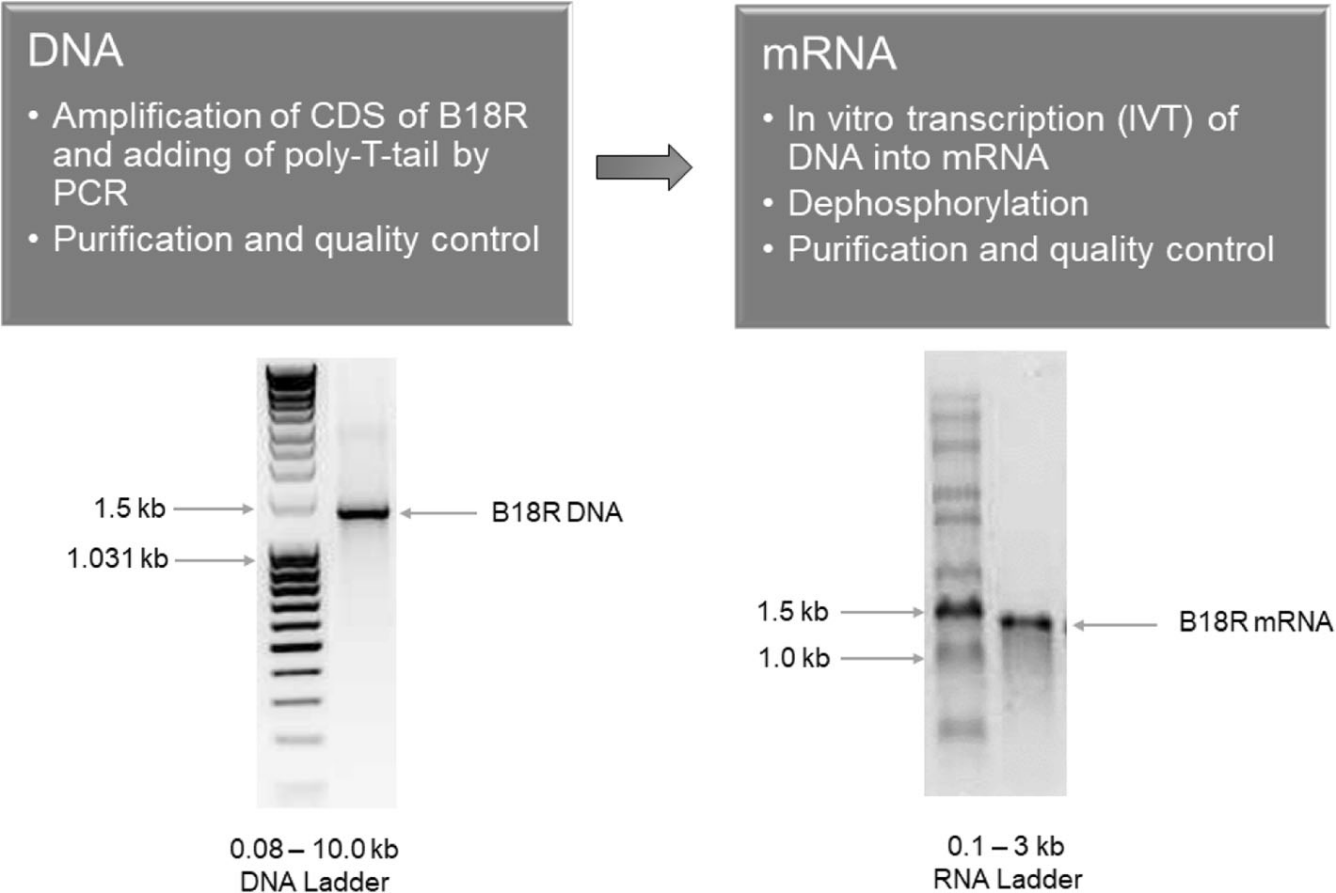
Schematic representation of the modified B18R mRNA synthesis. The coding sequence (CDS) of B18R was amplified from the pcDNA 3.3 vector using PCR. The amplified DNA was purified and the quality was examined by the detection of a single DNA band of approximately 1400 bases. Afterwards, the amplified DNA was used as template for the in vitro transcription (IVT) to generate the B18R mRNA. After the IVT, the transcribed mRNA was dephosphorylated, purified, and the quality of mRNA was proved using 1% agarose gel electrophoresis and the detection of one clear band with about 1400 bases

Using PCR, the CDS of B18R including 5’UTR, 3’UTR, and a poly-T-tail was amplified (Fig. 2). After the purification, the amplified DNA fragment was analyzed using 1% agarose gel electrophoresis. A clear band of approximately 1400 bases was detected. The subsequent IVT led to the production of the synthetic B18R mRNA with a length of about 1400 bases (Fig. 2).

### Proteome analysis for the detection of translated B18R protein in the cells

Fibroblasts were transfected with 1.5 μg eGFP mRNA. After 24 h, cells were lysed, 20 μg of the obtained protein was separated by SDS-PAGE, and proteome analysis was performed using LC-MS/MS. The analysis resulted in detection of 4 different peptides, which are specific for B18R protein (UniProtKB-Q9DUN2) (Table 1).

**Table 1:** Detected B18R specific peptide sequences in the B18R mRNA transfected cell lysate

Peptide Sequence	Proteins (UniProtKB)	PEP	Score	Intensity
ILTVLPSQDHR	Q9DUN2	4.43E-05	109.16	16,468,000
EINIDDIK	Q9DUN2	0.019146	64.711	11,597,000
IKNDIVVSR	Q9DUN2	0.0013751	85.622	1,828,500
YLCTVTTK	Q9DUN2	0.0070972	77.732	2,581,900

Abbreviation: *PEP* Posterior error probability

### Analysis of Mx1 gene expression after the transfection of cells with B18R mRNA

**Fig. 3:**
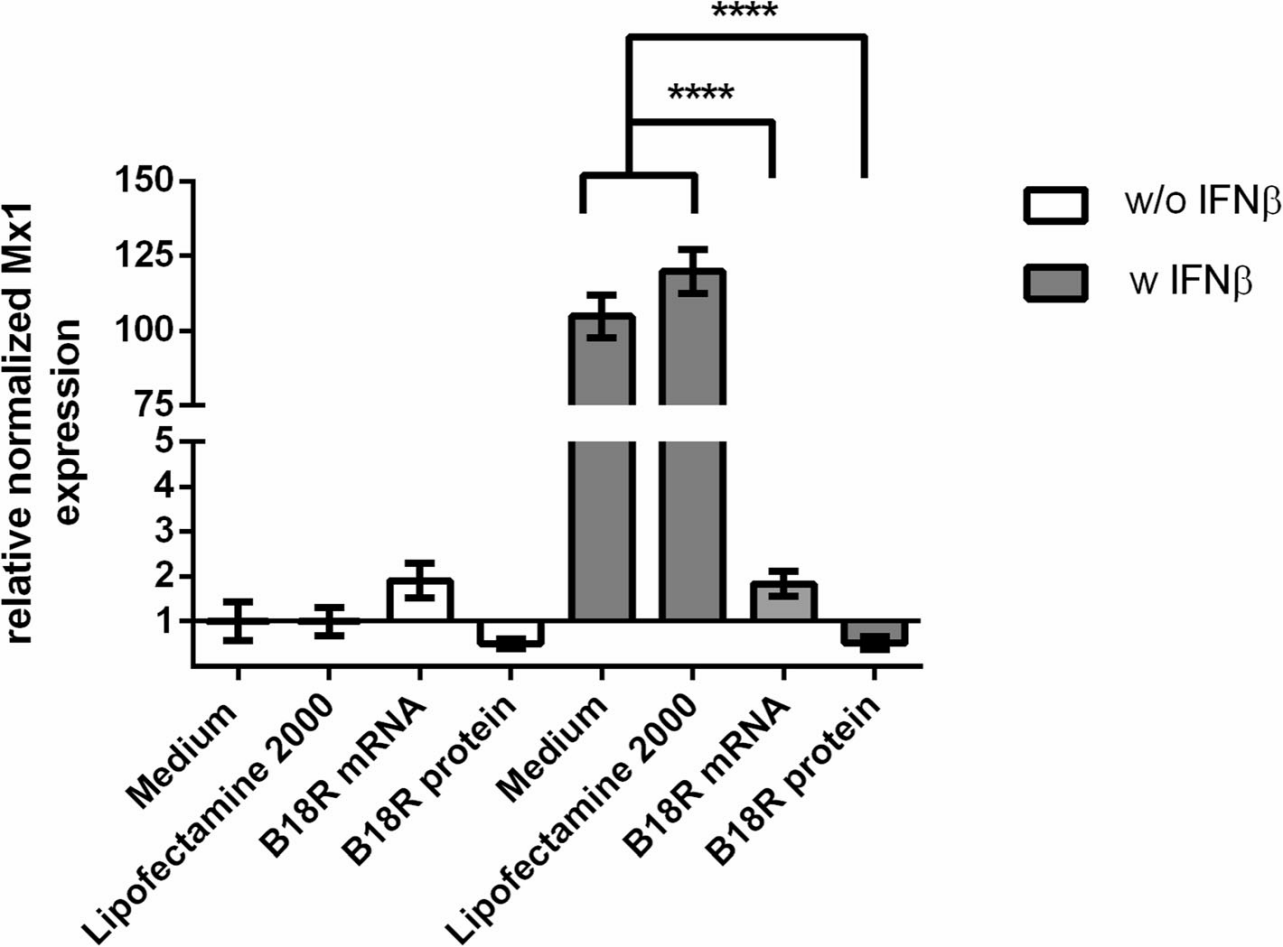
qRT-PCR analysis of Mx1 expression in fibroblasts transfected with synthetic B18R mRNA or incubated with 200 ng/ml B18R protein and the following stimulation with IFNβ. Fibroblasts were transfected with 1.5 μg B18R mRNA or incubated with 200 ng/ml B18R protein. After 24 h, cells were stimulated for 3 h at 37 °C and 5% CO_2_ with 5 ng/ml IFNβ_._ Subsequently, the Mx1 gene expression was analyzed using qRT-PCR. Results are presented as means ± SEM (*n* = 3). Differences were analyzed using one-way ANOVA following Bonferroni’s multiple comparison test. (****p < 0.0001)

To examine the ability of the delivered synthetic B18R mRNA to reduce the interferon-induced immune reaction by the production of B18R protein, fibroblasts were incubated after the B18R mRNA transfection with IFNβ. The expression of Mx1 transcripts was determined by using qRT-PCR. IFNβ stimulation of cells transfected with B18R mRNA or incubated with B18R protein resulted in a highly significant reduction of Mx1 expression, which showed the successful inhibition of IFNβ by the produced B18R protein in the cells (Fig. 3).

### Impact of B18R mRNA co-transfection on the translation of eGFP mRNA and cell viability

#### Expression of eGFP after the co-transfection of cells with B18R mRNA

**Fig. 4:**
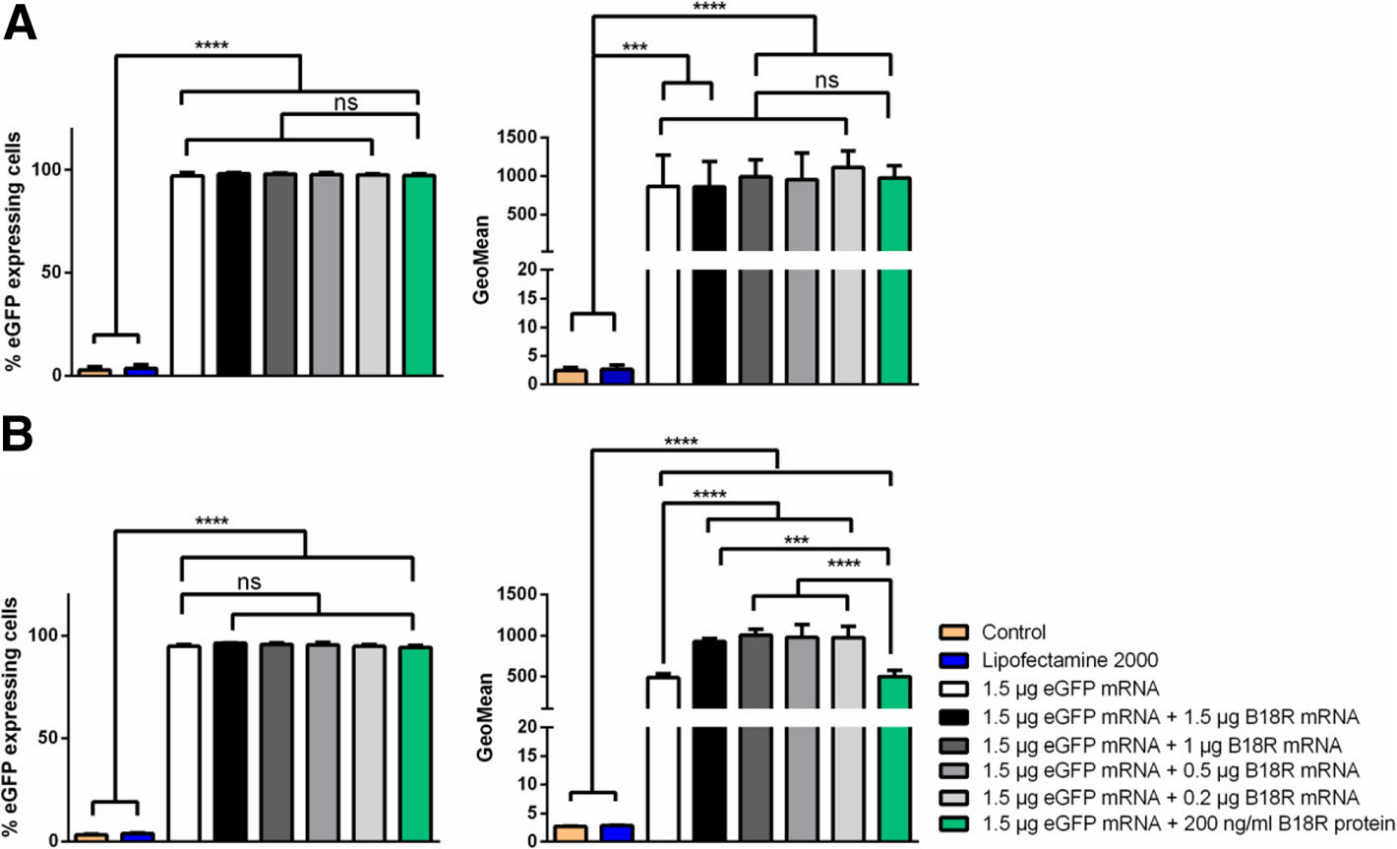
Investigation of eGFP expression after the co-transfection of fibroblasts with eGFP mRNA and different amounts of B18R mRNA using flow cytometry. 1 × 10^5^ fibroblasts were transfected for two following days with 1.5 μg eGFP alone or with 0.2, 0.5, 1, or 1.5 μg B18R mRNA. Cells treated with only Opti-MEM or Opti-MEM and the transfection reagent Lipofectamine^®^ 2000 served as negative controls. The eGFP expression was analyzed 24 h after (**a**) the first transfection and (**b**) the second transfection by flow cytometry. Results are presented as means ± SD (*n* = 3). Differences were analyzed using one-way ANOVA following Bonferroni’s multiple comparison test. (***p < 0.001, ****p < 0.0001). ns: not significant

To analyze the influence of B18R mRNA co-transfection on eGFP protein expression, 1 × 10^5^ fibroblasts were transfected simultaneously with 1.5 μg eGFP mRNA and 0.2, 0.5, 1, or 1.5 μg B18R mRNA. After 24 h, the cells were co-transfected again with the same amount of eGFP and B18R mRNA and incubated for 24 h. Flow cytometry analyses were performed 24 h after the first and the second transfection (Fig. 4). The co-transfection of cells with B18R mRNA showed no influence on eGFP expression 24 h after the first transfection (Fig. 4a). However, 24 h after the second transfection, cells co-transfected with eGFP mRNA and B18R mRNA resulted in a significantly higher eGFP expression compared to the cells transfected only with 1.5 μg eGFP mRNA (Fig. 4b). The eGFP expression in cells transfected with 1.5 μg eGFP mRNA and incubated with 200 ng/ml B18R protein was comparable to the eGFP expression in cells transfected with only 1.5 μg eGFP mRNA. Furthermore, increasing the amount of B18R mRNA from 0.2 to 1.5 µg did not result in significantly different eGFP expression.

**Fig. 5:**
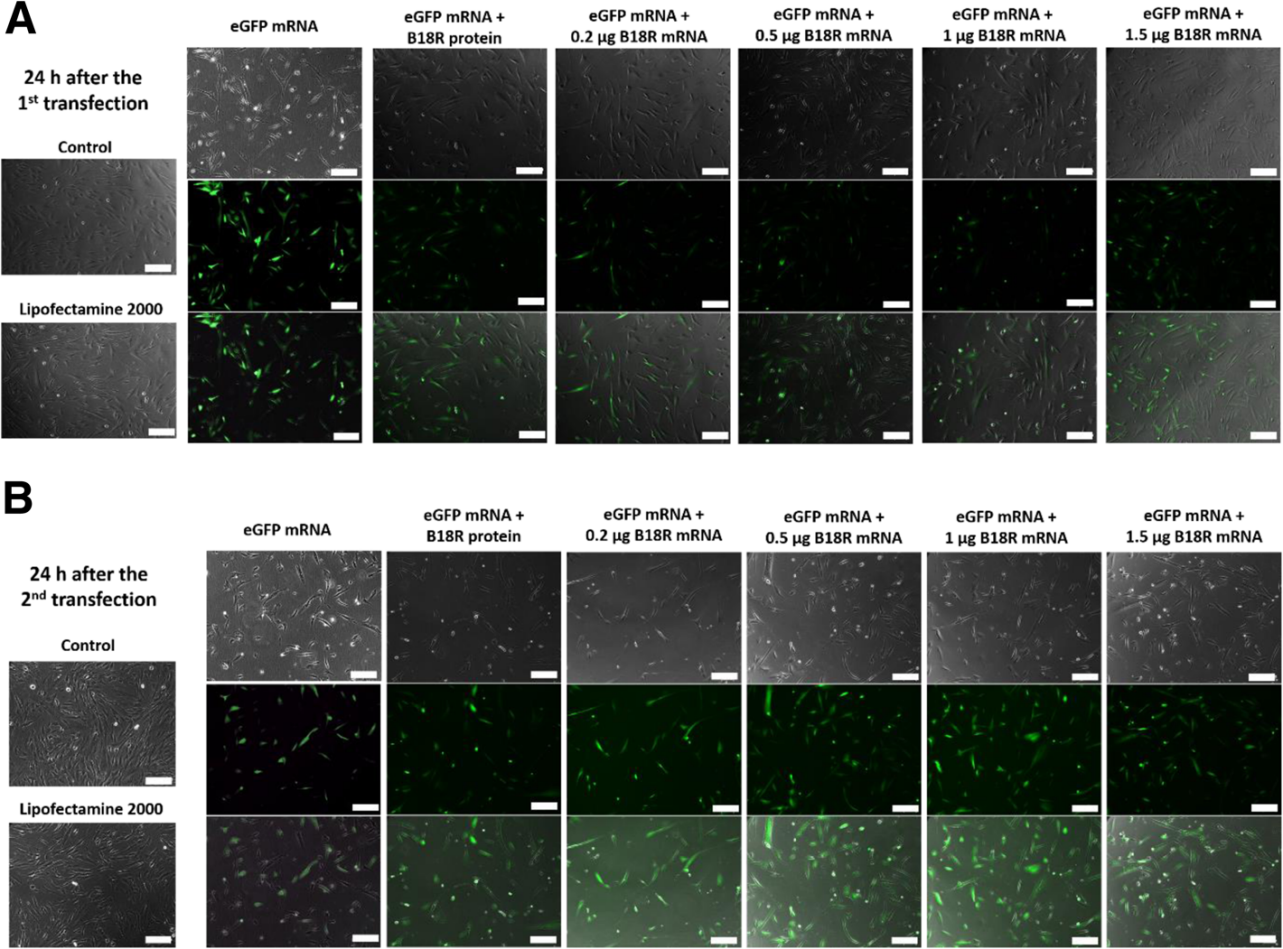
Investigation of eGFP expression after the co-transfection of fibroblasts with eGFP mRNA and different amounts of B18R mRNA using fluorescence microscopy. 1 × 10^5^ fibroblasts were transfected for two following days with 1.5 μg eGFP mRNA alone or with 0.2, 0.5, 1, or 1.5 μg B18R mRNA. The eGFP expression was analyzed by fluorescence microscopy 24 h after (**a**) the first and (**b**) second transfection. Scale bar corresponds to 200 μm

Additionally to the flow cytometry analyses, the expression of eGFP was also detected by fluorescence microscopy (Fig. 5). In accordance with the flow cytometry experiments, especially 24 h after the second transfection, increased expression of eGFP could be seen compared to the cells transfected with only eGFP mRNA or cells transfected with eGFP mRNA and incubated with B18R protein.

### Analysis of cell viability after the co-transfection of cells with eGFP mRNA and B18R mRNA

**Fig. 6:**
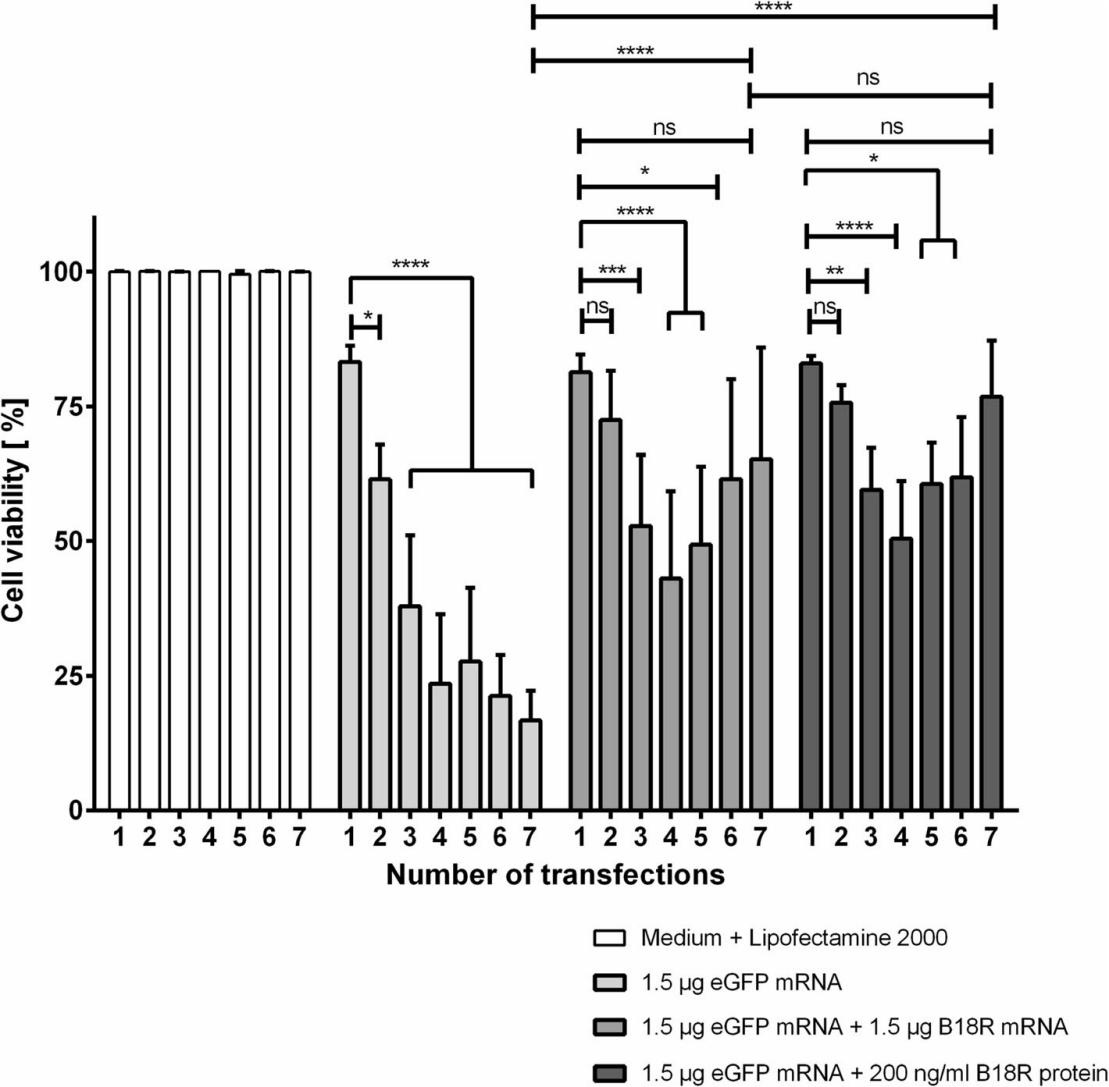
Impact of co-transfection with synthetic B18R mRNA on cell viability. Fibroblasts were transfected daily up to 7 days with 1.5 μg eGFP mRNA. Additionally, cells were transfected with 1.5 μg eGFP mRNA and 1.5 μg B18R mRNA or incubated with 200 ng/ml B18R protein after the transfection with 1.5 μg eGFP mRNA. The viability of cells after the Lipofectamine^®^ 2000 treatment was set to 100%. Results are presented as means ± SEM (*n* = 3). Differences were analyzed using two-way ANOVA following Bonferroni’s multiple comparison test. (*p < 0.05, **p < 0.01, ****p < 0.0001). ns: not significant

In order to investigate the viability of eGFP mRNA transfected cells in comparison to the eGFP mRNA transfected cells treated with B18R protein or co-transfected with B18R mRNA, 1 x 10^5^ fibroblasts were seeded per well of 6-well plate and transfected daily up to 7 days with 1.5 μg eGFP mRNA alone or with 1.5 μg B18R mRNA. Additionally, cells were transfected with 1.5 μg eGFP mRNA and incubated with 200 ng/ml B18R protein. Cells treated with Opti-MEM alone or Opti-MEM containing Lipofectamine^®^ 2000 served as controls. The cell viability was detected 24 h after each transfection using MTT assay (Fig. 6).

The repeated transfection of fibroblasts over 7 days resulted in a continuous significant decrease in cell viability. After 1 day, the cell viability of all treated cells was comparable. However, after the second transfection (Day 2), only eGFP mRNA transfected cells showed a significantly decreased cell viability. After 7 days of transfection, the cell viability of additionally B18R mRNA transfected (65.26% ± 20.64%) or B18R protein incubated (76.82 %± 10.42%) cells was significantly higher than the viability of only eGFP mRNA transfected cells (17% ± 5.52%). Furthermore, after 7 days, the viability of B18R mRNA co-transfected or B18R protein treated cells was not significantly different. A decrease of cell viability was detected until the 4th transfection, however, after the 5th transfection a continuous increase of cell viability was detected. After the 7th transfection, the cell viability was not significantly different than the detected levels after the 1st transfection.

## Discussion

In recent years, synthetic mRNA is applied for therapy [43], vaccination [44], and regenerative medicine [45]. While the synthetic mRNA-mediated activation of immune system is desired in the field of vaccination, in other applications, the activation of the immune system due to the delivery of synthetic mRNA is undesired. However, the immune reaction of cells to synthetic RNA molecules is a natural defense mechanism to protect them against viral infections. Although single mRNA transfections seem to be accepted by cells, the repeated daily transfection of cells with synthetic mRNA or the use of self-replicating RNA for example for the generation of iPSCs requires the suppression of IFN response to be able to perform the reprogramming procedure [37, 38].

B18R protein is an immune suppressor that interrupts the IFN response by binding and neutralizing the type I IFNs [34]. In the present work, we generated synthetic B18R mRNA and detected the translated B18R protein after the delivery of B18R encoding mRNA into the cells using MS. The co-transfection of synthetic mRNA transfected cells with B18R mRNA resulted in comparable IFN response inhibiting properties as the treatment with recombinant B18R protein, which was demonstrated by the reduced gene expression levels of anti-viral protein Mx1.

After a single transfection of cells with eGFP mRNA, no significant differences in eGFP expression were obtained in cells co-transfected with B18R mRNA or incubated with recombinant B18R protein compared to the cells transfected with only eGFP mRNA. However, after the second eGFP mRNA transfection, the co-transfection of cells with B18R mRNA resulted in an improved eGFP protein expression in contrast to the cells treated with B18R protein, which indicates that the B18R mRNA co-delivery has also a positive effect on translation of mRNA. This is possibly caused by reduced activation of the immune system and degradation of delivered synthetic mRNA.

After 7 consecutive eGFP mRNA transfections, significantly higher cell viability was detected in cells co-transfected with B18R mRNA or incubated with recombinant B18R protein compared to cells without B18R mRNA transfections or B18R protein treatment. In contrast, the repeated transfection of cells with only eGFP mRNA resulted in continuous decrease of cell viability. The cells treated with B18R mRNA demonstrated a similar cell viability as the cells treated with B18R protein. However, until 4th transfection, a continuous reduction of cell viability was detected also in B18R mRNA co-transfected cells and B18R protein treated cells. After the 5th transfection, a continuous increase in cell viability was measured. The reason therefore could be the required time for the blocking of all produced IFNs and the required time for the down-regulation of the expression of interferon-stimulated genes [46] and the adaptation of the cells. Possibly by starting B18R mRNA transfection or B18R protein treatment prior to the transfection with the desired mRNA, the protective effect could be reached immediately after the mRNA transfection of cells.

These results demonstrated that the cells transfected with synthetic mRNA could be simultaneously transfected with B18R encoding mRNA to reduce immune reactions and maintain the cell viability and protein expression. Thus, this method could be used for the long-term mRNA transfections and compared to the use of recombinant B18R protein, the application of B18R encoding mRNA is cheaper. Thereby, the transfected cells can produce their own IFN inhibiting protein. Particularly, in the field of iPSC generation using synthetic mRNAs, this method could be advantageous. Another possibility is also to transfect cells with B18R mRNA to obtain B18R conditioned medium, which can be used for the cultivation of mRNA transfected cells. Especially during the generation of iPSCs using self-replicating RNA, which requires only one transfection, the use of B18R conditioned medium could be beneficial.

## Conclusion

In this study, we demonstrated that the simultaneous transfection of cells with B18R mRNA can suppress the synthetic mRNA induced IFN response in cells. Therefore, B18R encoding mRNA was generated and the cells were simultaneously transfected with eGFP mRNA and B18R mRNA. B18R mRNA treatment significantly improved the cell viability and the production of eGFP protein in eGFP mRNA transfected cells. In future, B18R mRNA could be used together with reprogramming mRNAs to reduce and simplify the reprogramming procedure. Using B18R mRNA delivery, the immune activation can be efficiently reduced especially in cells repeatedly transfected with synthetic mRNAs. Thus, the co-transfection of cells with B18R encoding mRNA can be used as an alternative to the incubation with recombinant B18R protein.

## Abbreviations

ANOVA: Analysis of variance
ARCA: Anti-reverse cap analog
CDS: Coding sequence
DFG: German Research Foundation
DMSO: Dimethyl sulphoxide
FCS: Fetal calf serum
GAPDH: Glyceraldehyde 3-phosphate dehydrogenase
IFN: Interferon
IFNα: Interferon-α
IFNβ: Interferon-β
iPSCs: induced pluripotent stem cells
ISGs: IFN-stimulated genes
IVT: In vitro transcription
LC: Liquid chromatography
m5C: 5-methylcytidine
mRNA: messenger RNA
MS: Mass spectrometry
MTT: 3-(4,5-dimethylthiazol-2-yl)-2,5-diphenyltetrazoliumbromide
NF-κB: Nuclear factor κB
PCR: Polymerase chain reaction
PRRs: Pattern recognition receptors
qRT-PCR: Quantitative real-time polymerase chain reaction
RIG-I: Retinoic acid inducible gene I
SEM: Standard error of the mean
STATS: Signal transductors and activators of transcription
TLRs: Toll-like receptors
Ψ: Pseudouridine
